# A Potential Objective Sign of Central Sensitization: Referred Pain Elicited by Manual Gluteus Minimus Muscle Exploration is Coincident with Pathological Autonomic Response Provoked by Noxious Stimulation

**DOI:** 10.1155/2023/4030622

**Published:** 2023-02-02

**Authors:** Elzbieta Skorupska, Tomasz Dybek, Michał Rychlik, Marta Jokiel, Paweł Dobrakowski, Anna Szczerba, Daria Wotzka, Anna Jankowska

**Affiliations:** ^1^Department of Physiotherapy, Poznan University of Medical Sciences, Poznan, Poland; ^2^Faculty of Physical Education and Physiotherapy, Opole University of Technology, Opole, Poland; ^3^Department of Virtual Engineering, Poznan University of Technology, Poznan, Poland; ^4^Department of Traumatology, Orthopedics and Hand Surgery, Poznan University of Medical Sciences, Poznan, Poland; ^5^Psychology Institute, Humanitas University in Sosnowiec, Sosnowiec, Poland; ^6^Chair and Department of Cell Biology, Poznan University of Medical Sciences, Poznan, Poland; ^7^Faculty of Electrical Engineering, Automatic Control and Informatics, Opole University of Technology, Opole, Poland

## Abstract

Referred pain/sensation provoked by trigger points suits the nociplastic pain criteria. There is a debate over whether trigger points are related to a peripheral phenomenon or central sensitization (CS) processes. Referred pain is considered a possible sign of CS, which occurs probably mainly due to the abnormal activity of the immune and autonomic nervous systems. To confirm abnormal autonomic reactivity within the referred pain zone of active trigger points, a new diagnostic tool, the Skorupska Protocol® (the SP test®), was applied. The test uses noxious stimulation (10 minutes of dry needling under infrared camera control) as a diagnostic tool to confirm abnormal autonomic nervous system activity. A response to the SP test® of healthy subjects with referred pain sensations provoked by latent trigger points (LTrPs) stimulation was not explored before. The study aims at examining if LTrPs can develop an autonomic response. *Methods*. Two groups of healthy subjects, (i) gluteus minimus LTrPs with referred pain (*n* = 20) and (ii) control (*n* = 27), were examined using the SP test®. *Results*. Abnormal autonomic activity within the referred pain zone was confirmed for all analyzed LTrPs subjects. 70% of control subjects had no feature of vasodilatation and others presented minor vasomotor fluctuations. The size of vasomotor reactivity within the referred pain zone was LTrPs 11.1 + 10.96% vs. control 0.8 + 0.6% (*p* < 0.05). *Conclusions*. Noxious stimulation of latent TrPs induces abnormal autonomic nervous system activity within the referred pain zone. The observed phenomenon supports the concept of central nervous system involvement in the referred pain patomechanizm.

## 1. Introduction

In the last decade, a growing interest in myofascial pain syndrome (MPS), characterized by sensory, motor, and autonomic symptoms provoked by trigger points (TrPs), is observed. Importantly, some subtypes of MPS described by Travell and Simons [1, 2] (e.g., cervical, thoracic, and lumbar pain related to TrPs) were included in the 11th Revision of the International Classification of Diseases [3]. Trigger points are defined as hyperirritable painful spots within taut bands of skeletal muscles that are painful on compression, stretch, overload, or contraction of the tissue which usually respond with referred pain that is perceived distant from the spot. Active and latent TrPs are distinguished. The active form of differentiation is based on spontaneous, recognizable pain mainly [4].

Currently, pain specialists hotly debate whether TrPs are a central or peripheral phenomenon and what are the MPS pain pathomechanisms [5]. One hypothesis states that the central sensitization (CS) processes is involved in TrPs development. This theory is supported by two phenomena, such as referred pain provoked by TrPs (secondary hyperalgesia) together with lower pressure pain thresholds within the muscle fiber affected by TrPs (primary hyperalgesia) [6–8]. The sympathetic activity depended on the referred pain presence was also demonstrated. This additionally confirms the CS involvement in TrPs development [9–11]. The other theories postulate that trigger points are just nociceptive peripheral sensation, denying their clinical importance or even questioning their existence [12]. Functional impairments of the autonomic and immune systems are indicated as a possible reason of the processes of central sensitization [13, 14]. The most commonly accepted hypothesis of TrPs development was presented by Simons [15]. The authors proposed 5-6 stages of the TrPs development. Still, they pointed out the last stage as the one which might result in development/maintenance of TrPs symptoms and induced by the autonomic nervous system (ANS) activity.

To examine abnormal autonomic activity within referred pain and possibly to confirm TrPs objectively, a new diagnostic method, the Skorupska Protocol (SP) test®, was established lately. The test is based on the observation of noxiously provoked amplified vasomotor reactivity coincident with the referred pain zone established previously by Travell and Simons [1, 2, 10, 16].

The abnormality of the ANS response-observed during the SP test® is based on the fact that, in normal conditions, the noxious stimulation of nociceptors in skeletal muscles induces only a local vasomotor reaction limited to the area of the noxious stimulation. On the contrary, the stimulation of the active TrPs within the gluteus minimus provoked distant vasomotor reactivity spreading down the lower extremities. Furthermore, according to the current knowledge about the physiology of the ANS integrative functioning, the elicitation of vasomotor reactivity in the lower extremities demands ANS activity at the level of the spinal cord and/or hypothalamus [17]. This fact further supports the hypothesis of the central nervous system's involvement in the TrPs patomechansim. The amplified vasomotor phenomenon within the thigh and calf, on the low back pain patients, confirmed by SP test®, occurred probably due to a pathological spinal reflex characteristic of TrPs exclusively, i.e., the so-called twitch response that is linked to the ANS [18]. It is worth mentioning that there is no link between vagal control/regulation and gluteal blood supply; this provides further evidence for the abnormal ANS involvement.

Still, it must be remembered that referred pain is also typical for asymptomatic, pain-free healthy subjects with latent TrPs, and it can be provoked by mechanical muscle pressure or needle irritation [19, 20]. A recent study by Ambite-Quesada et al. supports the involvement of central sensitization in referred pain from latent trigger points by applying the quantitative sensory testing technique [21]. Nevertheless, based on the assumption that referred pain is a sign of central sensitization, it is necessary to reveal if latent trigger points are likely to provoke pathological autonomic reactivity within the referred pain zone just like active trigger points.

Thus, the aim of the study was to examine referred pain from latent trigger points towards the presence of pathological autonomic reactivity among healthy subjects.

## 2. Material and Methods

The study was approved by the Ethics Committee of the Poznan University of Medical Sciences (resolution number 689/20) and was conducted in accordance with the Declaration of Helsinki. Before data collection, all subjects gave written informed consent to participate in the study. A detailed description of all examinations was provided to the participants who had the right to refuse the SP test® performance and withdraw from the study at any time without penalty.

### 2.1. Participants

Seventy-five healthy subjects were assessed for eligibility.

The inclusion criteria were as follows: (1) for LTrPs subjects: general good health condition (pain-free, without any medical diagnosis of permanent disease or surgery in the past, current fever, or infection), age between 20 and 60 (inclusive), both lower limbs present, and latent trigger points within the gluteus minimus muscle that developed the referred pain pattern due to muscle pressing; (2) for the control subjects: general good health condition, age between 20 and 60 (inclusive), both lower limbs present, and the lack of (i) latent trigger points within the gluteus minimus muscle or (ii) local or referred unrecognized pain after cross-fiber flat palpation of the gluteus minimus/medius/maximus muscle.

The key exclusion criteria were as follows: previous back surgery, spinal tumors, scoliosis, and pregnancy.

### 2.2. Methods

All subjects enrolled in the study were diagnosed towards latent trigger points presence by two independent therapists experienced in myofascial pain diagnosis. Then, the SP test® was performed using a thermovision touchless camera NEC-AVIO TVS-200EX (measurement error ± 2% in the range of the temperatures between 0–100 Celsius degree) with 8–14 *μ*m wave band, temperature resolution better than 0.080°C, sensitivity of 80 mK, and working in real time. The camera was equipped with a high-speed (60 Hz) uncooled FPA 320 × 240 (H × V) pixels VOx (vanadium oxide) microbolometer. For thermal images analysis, the specialist program “Thermography Studio 2007 Professional” was used.

#### 2.2.1. Diagnostic Criteria for Trigger Point Presence

According to Travell and Simons [1, 2], the taut band (one of the essential clinical criteria) is unlikely to be palpated for the gluteus minimus muscle because it lies deeper than the gluteus maximus and gluteus medius muscles. However, TrPs spot tenderness can be clearly localized. Additionally, the referred pain pattern is more likely to be observed when the needle encounters TrPs rather than when sustained pressure on the tender spot is applied.

Thus, the diagnosis of latent TrPs within the gluteus minimus muscle was based on Travell and Simons [2] diagnostic criteria, supported by the confirmation of referred pain elicited by deep snapping palpation through the gluteus minimus zone. Next, healthy subjects were rediagnosed using the new Delphi criteria for latent trigger points [22]. The criteria included: local or referred unrecognized pain occurrence, painful sensations only when palpated, the lack of reproduction of symptoms experienced by the patient, and no recognition of the symptoms previously caused by cross-fiber flat palpation [22]. All subjects who tested positive for both clinical criteria were then examined using the SP test® by the same experienced myofascial therapist who had diagnosed the patients before.

#### 2.2.2. The SP Test® Description

A new diagnostic tool, the Skorupska Protocol®, was established to examine abnormal autonomic activity within the trigger points referred pain. The method has undergone the validation and reliability process [23]. The test allows the registration of amplified vasomotor reactivity (vasodilatation and/or vasoconstriction) in the patient's daily complaint area, coincident with referred pain from trigger points located in the tested muscle. The SP test® provides information about the following: (i) the size of the observed phenomenon expressed as a percentage of the examined part of the body; (ii) the changes in the average temperature increase within the observed phenomenon −Δ$\bar{T}$°; (iii) the time interval within the examination when the autonomic phenomenon occurred.

The muscle examination is based on the noxious stimulation (10 minutes of fast-in-fast-out dry needling followed by 6 minutes of further observation of the patient at rest) under infrared thermal (IRT) camera control to detect the expected referred pain zone in the examined muscle. The two SP test® phases (stimulation–10′ and observation–6′) are consistent with active dynamic thermography protocol demands [24]. To determine if noxious stimulation of the muscle provoked abnormal autonomic activity within the referred pain zone defined for the examined muscle by Travell and Simons [2], the analysis of a series of 320 thermal pictures was performed. The time interval between the consecutive thermograms was 3 seconds, and the amplified vasomotor reactivity above/under the cutoff point was recorded. The cutoff point is the smallest subarea of the highest/lowest temperature before stimulation (state at rest).

#### 2.2.3. The Terms for the Confirmation of Abnormal Autonomic Nervous Activity Related to TrPs

Amplified vasomotor activity is confirmed if the SP test® provokes the development of a new thermal subarea above (amplified vasodilatation) or under (amplified vasoconstriction) the cutoff point within the expected referred pain zone. The second condition is that this new subarea provoked by the noxious TrPs stimulation is characterized by average temperature changes of more than 0.3°C, compared to the average temperature of the subarea defined as the cutoff. To calculate the size of the autonomic phenomenon and Δ$\bar{T}$° changes, the automated segmentation of all collected thermograms towards the presence of specific subareas above/under the cutoff was performed by MATLAB. Based on the occurrence of the new thermal subarea above/under the cutoff, it is possible to examine if the noxious stimulation of given TrPs provokes amplified vasomotor reactivity within the referred pain zone.

#### 2.2.4. A Short Description of the SP Test®

(1) Trigger points examination according to palpatory diagnostic criteria.
(2) Examination according to a typical IRT protocol to evaluate if a patient additionally presents features of the neuropathic pain pathomechanism (possible mixed pain syndrome), i.e., side-to-side comparison under infrared thermal camera control to examine the pain region toward a temperature decrease of more than 0.5°C.
(3) The SP test®, i.e., the IRT-controlled examination of referred pain from the analyzed muscle:
  - Noxious, nociceptive muscle stimulation under infrared thermal camera control of the area with expected muscle-referred pain (10 minutes), where noxious stimulation is fast-in-fast-out dry needling of TrPs or two areas that were the most tender to pressure within the examined muscle
  - Poststimulation resting phase, i.e., further thermal observation of the patient at rest (6 minutes)
(4) MATLAB analysis of the collected data to calculate two SP test® parameters: (i) the size of the observed vasomotor phenomenon (AURP cutoff) and (ii) the average temperature increase within the observed phenomenon (Δ$\bar{T}$°).
(5) The results are based on the segmentation of each thermogram and the calculation of both parameters for the observed anatomical body parts. The first parameter, called autonomic referred pain (AURP cutoff), is the size of the subarea with vasomotor reactivity expressed as a percentage of the observed anatomical area. This parameter reflects a limited subarea, whose size is defined by a region with a temperature that is not registered for the patient before the stimulation (cutoff). The second parameter is the exact value of the average temperature changes (Δ$\bar{T}$°) within the observed phenomenon.
(6) Final statistical analysis of SP test® results and confirmations of the autonomic phenomenon measured every 3 seconds of the procedure.

**Figure 1:**
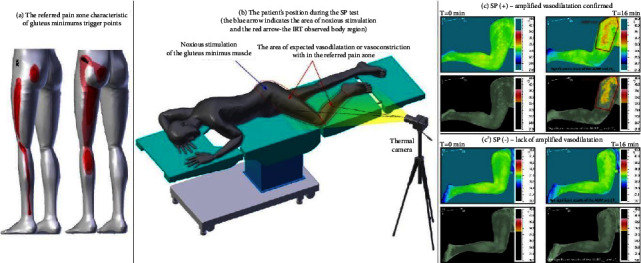
An illustration of the SP test® applied to latent trigger points of healthy subjects: (a) the referred pain zone characteristic of gluteus minimus trigger points; (b) the patient's position during the SP test® (the blue arrow indicates the area of gluteus minimus noxious stimulation and the red arrow shows the body region, that is, coincident with the gluteus minimus referred pain zone, and that was observed for possible autonomic reactivity using an infrared thermal camera; (c) an example of an LTrPs patient's response to the SP test®; (c′) an example of a healthy subject's response to the SP test®.

An illustration of the SP test® protocol and an example of the test results are shown in Figure 1.

#### 2.2.5. MATLAB Protocol Development

**Figure 2:**
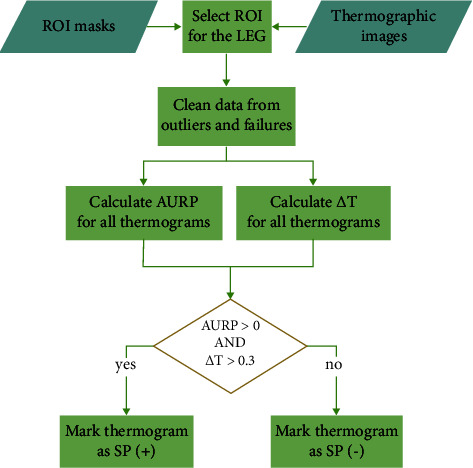
The procedure applied for calculating the final results of the SP test®.

A detailed protocol description, the method's validation, reliability, and the MATLAB procedure established for the SP test® were presented previously [10, 16, 23]. The procedure for the calculation of the final the SP test® results is additionally presented in Figure 2.

**Figure 3:**
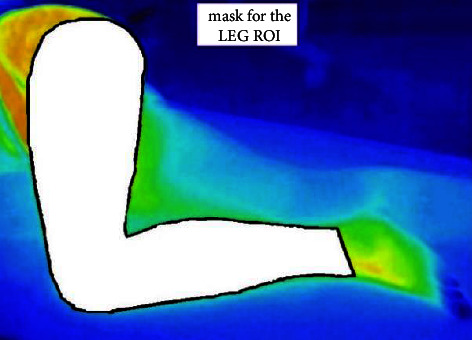
The mask applied for the ROI determination in a representative thermogram.

In the first step, the region of interest (ROI) is determined for every subject based on the initial thermogram and a manually created ROI mask for the thigh and calf. A representative ROI mask is depicted in Figure 3. The masks were created by hand and further used during the automatic procedure applied for ROI detection. In the next step, all gathered data underwent the cleaning procedure, where outliers and faulty thermograms were deselected from the final data set.

Based on the final data set of AURP cutoff and Δ$\bar{T}$° values, the final results of the SP test® were calculated. For each thermogram, the AURP cut-off value was calculated as a percentage of the LEG surface with a temperature greater than the maximum temperature registered for the subject before the stimulation and the Δ$\bar{T}$° value was calculated as a change in the average temperature. The first image registered before the stimulation for the subject at rest was used as a reference point for both parameters.

In the final step, the SP test® results were provided. To confirm autonomic phenomenon occurrence (the SP test® positive results)–the size of the possible amplified vasomotor reactivity was calculated for each thermogram based on the confirmation of two SP test® parameters: (i) AURP cutoff occurrence within the observed area, and (ii) Δ$\bar{T}$° value greater than 0.3°C.

### 2.3. Statistical Analysis

For an exact test at a significance level of 0.05 with a beta power of 0.95, the lowest possible sample size is *n* = 19. Exact two-tailed Mann–Whitney *U* tests with corrected ties were performed to assess the differences between the gluteus minimus LTrPs patients (*n* = 20) and healthy controls (*n* = 27). The tests were applied to compare the final SP test® results between the aforementioned groups and additionally for both SP test® parameters (AURP cutoff and Δ$\bar{T}$°) separately. To check the significance of the p values, a post hoc Dunn test was performed. Due to the multiple comparison problem, the aforementioned test was corrected using the Holm–Sidak procedure. The Dunn test was prepared using the Dunn. test package in *R*. All values, figures, and tables in the text are expressed as the means ± standard deviations (SD) or as quartiles with the median. The significance level was set for all tests at *p* <  0.05. To obtain relevant sample size G *∗* Power 3.1.9.7 calculator was used. The effect size was fixed at the 0.35 level. Statistical analysis was performed using IBM SPSS Statistics version 26 and MATLAB version R2021.

## 3. Results

### 3.1. Subjects Examined by the SP Test®

Twenty-five of the subjects assessed for the SP test® eligibility were excluded: seven (*n* = 7) declined to participate and eighteen (*n* = 18) did not meet the inclusion criteria. Twenty subjects with latent gluteus minimus trigger points (LTrPs) (*n* = 20) and thirty healthy subjects with no gluteus minimus trigger points (control) (*n* = 30) were included in the study. During the SP test®, three subjects (*n* = 3) from the control group reported referred pain sensations in the referred pain zone typical for the gluteus minimus muscle. Thus, these three subjects were excluded and the control group consisted of twenty-seven healthy subjects (*n* = 27).

### 3.2. General Results of the SP Test® Examination

Amplified vasodilatation (necessary for a positive results of the SP test®) was confirmed for the LTrPs subjects exclusively. As many as 70% of the control group showed no vasomotor reactivity. The remaining 30% of the control subject (*n* = 9) presented small temperature fluctuations. The size of amplified vasodilatation in the LTrPs group was significantly bigger compared to the control subjects who presented small vasomotor reactivity (*p* < 0.05; Mann–Whitney *U* test). The results obtained at two measurement points of the test, i.e., (i) the end of the noxious stimulation and (ii) the end of the poststimulation observation phase of the test and are shown in Table 1.

**Figure 4:**
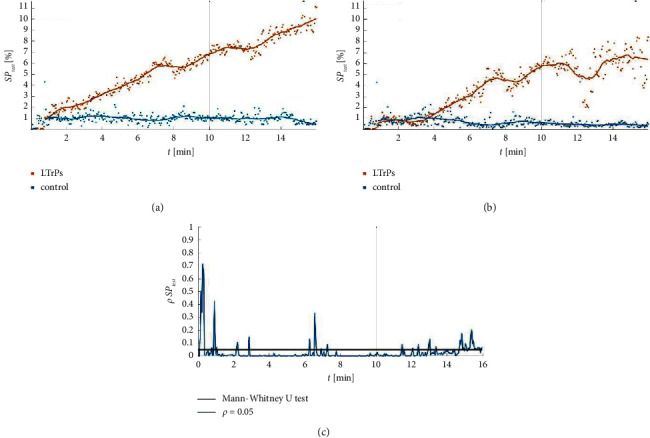
MATLAB trends of the SP test® results depend on the latent trigger points presence and the SP test® phase: (a) median value, (b) average value, and (c) Mann–Whitney *U* test results. The LTrPs subjects presented abnormal autonomic activity that was confirmed by amplified vasodilatation. The MATLAB trends showed the development of the percentage size of amplified vasodilatation spreading in the lower leg in time. The significant difference between both groups stabilized around 2′ of the noxious stimulation, but in 4′ the size of the amplified vasodilatation had a tendency to an intensive increase. The control showed stable thermal fluctuations during the whole procedure.

A key characteristic of the observed autonomic phenomenon among SP test® positive subjects was as follows: (i) the LTrPs subjects presented its further development after the end of the noxious stimulation with the maximum percentage size of amplified vasodilatation reached at 15′43′′ (observation phase of the test); (ii) amplified vasodilatation seen in the LTrPs subjects lasted from 1′00′ to 16′00′′ (93.8% of the test duration) (*p* < 0.05). The MATLAB trends that present the size of the vasomotor response to the SP test® measured every three seconds are shown in Figure 4.

### 3.3. Results of the SP Test® Examination Depending on the Anatomical Part of the Leg

All LTrPs subjects (100%; *n* = 20) developed a response in the thigh, and some of them additionally presented amplified phenomenon in the calf (20%; *n* = 4). For the control subjects (*n* = 9) who presented vasomotor reactivity, a series of small hot spots was observed in the thigh (*n* = 9) and calf (*n* = 7). Due to the small number of subjects presenting a vasomotor response on the calf, the statistical analysis of the results characteristic of this group was not possible.

The SP test® results for the thigh were as follows: (i) at the end of the noxious stimulation (10′ of the SP test®): LTrPs median 8.7 (0.5, 35.8), 10.1 ± 8.98% vs. control median 1.0 (0.1, 6.6), 2.3 ± 2.37%, and (ii) at the end of the poststimulation observation phase (16′ of the SP test®): LTrPs median 13.3 (0.07,45.59), 16.47 ± 15.8% vs. control median 2.4 (0.3, 8.3), 3.2 ± 2.8%.

### 3.4. The Results of the Average Temperature Changes in the Observed Lower Leg According to Conservative Medical Thermography Assessment

**Figure 5:**
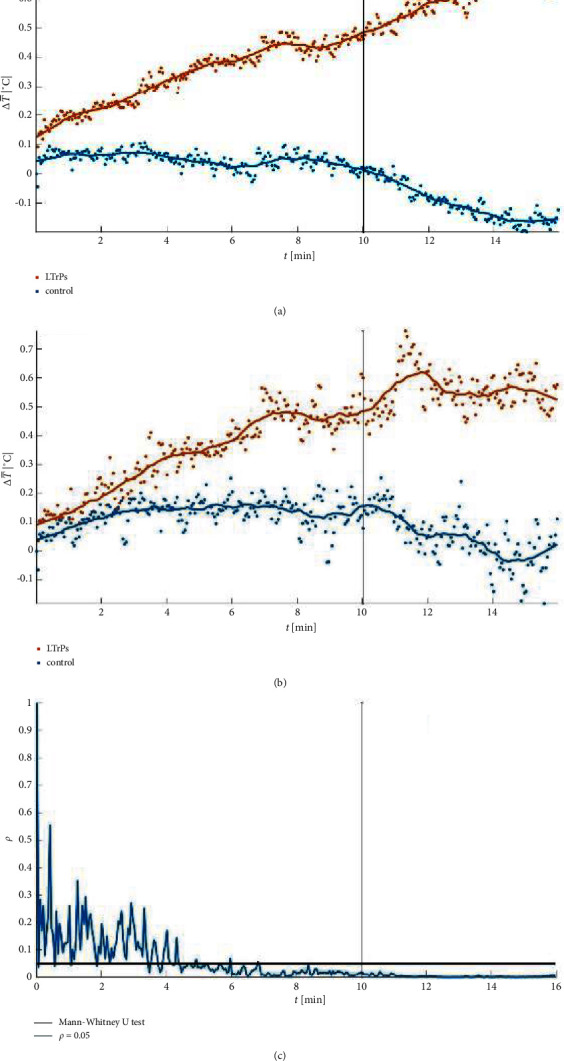
MATLAB trends of the development of the average temperature changes compared to the cutoff point as observed during the SP test® depend on the examined group and time of the procedure 4: (a) median value, (b) average value, and (c) Mann–Whitney *U* test results. The control group was characterized by the temperature decrease, which confirmed the lack of abnormal autonomic phenomenon^*∗*^ in this group (^*∗*^the conditions of the positive the SP test® for vasodilatation: (i) development of a new thermal subarea above the cutoff; (ii) ΔT increase of more than 0.3°C as compared to the cutoff point).

The average temperature changes (for LTrPs (*n* = 20) vs. control (*n* = 27)) were measured in the thigh and calf, and were calculated as one region of interest (ROI). Δ$\bar{T}$° results for both examined groups were as follows: (i) at the end of the noxious stimulation (10′ of the SP test®): LTrPs (*n* = 20) median 0.6 (−0.4, 1.2), 0.5 ± 0.47°C vs. control (*n* = 27) median 0.08 (−1.1, 1.07), 0.02 ± 0.56°C (*p* < 0.05; Mann–Whitney *U* test, and (ii) at the end of the poststimulation observation phase (16′ of the SP test®): LTrPs median 0.6 (−0.02, 1.4), 0.7 ± 0.44°C vs. control median 0.11 (−1.3, 0.8), −0.12 ± 0.61°C (*p* < 0.05; Mann–Whitney *U* test. The development of Δ$\bar{T}$° observed for the examined groups every three seconds of the SP test® are shown in Figure 5.

## 4. Discussion

The SP test® was developed to diagnose abnormal autonomic activity within the trigger points (TrPs) referred pain zone as a possible sign of the autonomic nervous system involvement in the referred pain/sensation phenomenon [16, 23]. Until now, this reaction has been confirmed for patients with active trigger points characterized by amplified vasodilatation that is coincident with the referred pain zone.

The present study was aimed at examining whether gluteus minimus latent trigger points (LTrPs) can develop an abnormal autonomic response to the SP test®.

All subjects with referred pain that was provoked from gluteus minimus LTrPs were characterized by amplified vasodilatation. The SP test® revealed that the LTrPs subjects presented a constant increase in the amplified vasodilatation size from the first minute to the end of the test. Importantly, the biggest size was reached during the poststimulation observation phase of the test. The majority of the control group revealed no signs of vasomotor reactivity. Three out of ten subjects presented small vasomotor reactivity during the test. Still, these values were significantly smaller compared to the ones presented by the LTrP subjects (*p* < 0.05). Moreover, control subjects presented a decrease in the average temperature of the observed area when compared to the cutoff point established for the SP test®. This indicated the lack of amplified vasodilatation. The analysis of the SP test® results depended on the anatomical parts, and it showed that only a few subjects from the LTrPs group presented amplified vasodilatation in the calf, which reflects intersubject variability in referred pain occurrence. The analysis of the average temperature changes in the observed region indicated that the poststimulation observation phase was characterized by a further temperature increase among the LTrPs subjects, contrary to the controls who presented the average temperature decrease.

The pathological autonomic reactivity of latent TrPs is similar to characteristic of active trigger points, which were previously examined using the SP test® [10, 16]. However, the phenomenon observed in case of latent TrPs analysis differed in size. The latent form was characterized by a smaller size of the observed amplified vasodilatation and a lower temperature increase within the referred pain zone that occurs due to noxious stimulation. For comparison, as a result of gluteus minimus active trigger points stimulation, sciatica patients with TrPs presented amplified vasodilatation with the size of more than 30% of the observed area and a Δ$\bar{T}$° increase above 1°C [25]. Furthermore, the results of the present analysis are contrary to some of other available studies reporting attenuated vasoconstriction due to glutamate injection to trigger points or some vasomotor changes not detectable by infrared thermal (IRT) imaging [13, 14]. This discrepancy can be explained by a different type of noxious stimulus used during the SP test®, which involved direct long-lasting mechanical nociceptive muscle stimulation instead of the chemical one, achieved by glutamate injection [26, 27]. This hypothesis is consistent with Nickel et al. study [26], which showed that the autonomic nervous system reactivity depends on the time and intensity of the noxious stimulus, but not the clinical state or pain level. Additionally, we suggest that the local twitch response evoked by dry needling used as noxious stimulus during the SP test® probably may have essential meaning for provoked amplified vasomotor response into referred pain zone. As stated above, amplified vasodilatation within the lower leg due to nociceptive muscle stimulation demands the ANS activity at the level of the spinal cord and/or hypothalamus. Thus, the twitch response defined as a possible pathological spinal cord reflex that leads to autonomic and motor effects in the referred pain zone, can explain the changes observed evoked by the SP test® [2, 15, 28].

Though the most important result of the study is observation, we found that latent TrPs, similarly to active TrPs, provoked amplified vasomotor reactivity within the referred pain zone. The vasomotor reactivity provoked by the SP test® can be explained by the activation of the nonnoradrenergic vasodilator system, which affected the processes of reflex cutaneous vasoconstriction and vasodilatation and reflected a temporary autonomic nervous system (ANS) imbalance within the referred pain zone [29, 30]. Furthermore, the observed phenomenon is unique to TrPs-related referred pain [7, 21, 31]. The IRT-controlled needle stimulation of an acupoint has been shown to result in vasodilatation spreading a maximum of 5–10 centimeters from the stimulation point or has failed to visualize any reactions apart from technical artifacts [32, 33]. Ten-minute dry needle stimulation of soft tissue can explain the dynamic and extensive autonomic response observed in the present study.

The concept of nociplastic pain related to muscles is based on the link between the TrPs-referred pain mechanism and the central sensitization (CS) process that has been postulated in the literature [10, 21]. It has been hypothesized that the process of CS involvement in muscles is initiated by a brief burst of C-fiber activity followed by nociceptor activity that provokes the excitability of central nociceptive neurons in the cortex, brain stem, trigeminal nucleus, and spinal cord [34]. In this process, the ANS dysregulation is indicated as one of the main causes of the central sensitization phenomenon development and/or maintenance [13, 14]. Moreover, central sensitization is characterized by secondary hyperalgesia, allodynia, and/or the presence of increased temporal summation of pain [35]. Temporal summation manifests itself as an increasing response to repeated nociceptive stimulations within the same receptive field [6]. The analysis of both the SP test® diagnostic parameters (namely, the size of the amplified vasomotor reactivity and ΔT increase higher than >0.3°C) measured every 3 seconds revealed the fluctuating and increasing in time character of the observed phenomenon. Additionally, a further increase in the SP test® parameters seen during the observation phase can be a mark of the temporal summation characteristic of CS. This supports the hypothesis that TrPs more probably represent a central phenomenon not a peripheral sensation. However, the concept of central sensitization processes has some gaps, especially when both active and latent TrPs are considered. On the one hand, CS is characteristic of chronic pain patients. On the other hand, CS is associated with a family history of pain, high psychological comorbidity, increased sensitivity to nonpain sensory stimuli, and a high number of chronic overlapping pain conditions. It is worth noting that the beginning of the symptoms is associated with puberty [36].

In addition to mention above hypothesis, there is also a new concept by Harte et al. proposing that two types of CS should be distinguished, namely, top-down and bottom-up. The first one is characterized by a greater number of severe sensations typical, for example, fibromyalgia, where the primary problem likely originates from the supraspinal structures and which symptoms are irreversible. The second type is defined as a possibly to reverse the process. This bottom-up type of CS is characterized by pain due to an excess noxious peripheral input that eventually sensitizes the central nervous system to the point of perceiving pain. Harte et al. [36] stated, that over a time, pain is perceived even when there is no peripheral drive. Generally, the bottom-up CS subtype is indicated as a lower burden. Based on that statement, it can be hypothesized that TrPs might be categorized as bottom-up. However, if we consider that CS is characteristic of patients with pain, the referred pain provoked by latent TrPs can be categorized as nociplastic one, which is a broader term than CS.

A potential role of the autonomic nervous system involvement in nociplastic pain (central sensitization) and TrPs development has been indicated by other authors [6, 28, 29]. Even though only gluteus minimus TrPs were examined using the SP test®, it can be assumed that other muscles with TrPs will react similarly. The fact that latent TrPs presented a pathological autonomic phenomenon just like active TrPs allows us to believe that the autonomic nervous system measurement can possibly play a crucial role in an objective diagnosis of nociplastic pain related to muscle. The idea that the SP test® can possibly become a new diagnostic method for the objective confirmation of nociplastic pain related to TrPs, understood as a subtype of central sensitization and probably categorized as bottom-up, seems worth addressing [36, 37]. Further studies considering the SP test® application to other muscles with both types of trigger points are recommended to support the concept that referred pain can be classified as the source of nociplastic pain related to trigger points.

### 4.1. The Clinical Implications of the Study

The SP test® allows the confirmation of both active and latent gluteus minimus TrPs. Thus, it might be presumed that the test can be used to objectively confirm referred pain in other muscles. This provides an opportunity for extensive clinical studies towards nociplastic pain involvement in patients with musculoskeletal pain disorders. However, further studies considering the SP test® response of other muscles that provoke referred pain are necessary.

### 4.2. Limitation of the Study

The main limitation of the study is the fact that the SP test® is a 10-minute painful protocol. The dry needling technique used as a noxious stimulus is widely applied as a therapeutic tool in clinical practice. The extended time of dry needling stimulation, above the clinical recommendation, was applied for diagnostic purposes only. All of the patients withstood the SP test® but they confirmed unpleasant sensations. Moreover, the therapist who performed dry needling in the present study was not blinded to the trigger point diagnosis, which could have biased the results to some extent.

## 5. Conclusions

Noxious stimulation of latent TrPs provoked abnormal autonomic nervous system activity within the referred pain zone. The observed phenomenon supports the concept of central sensitization related to trigger points. Further studies towards the autonomic response of other muscles with trigger points are recommended.

## Figures and Tables

**Table 1:** The SP test® results of the healthy subjects depend on the latent trigger points presence.

The SP test® gluteus minimus muscle	Parameter description	The size of amplified vasodilatation, which covered lower leg (%)	
		LTrPs	Control
End of stimulation (10′ of the test)	Median (Q1, Q3)	5.8(0.09, 24.6)^*∗*^	0.6(0.03, 3.1)^*∗*^
	Average ± SD	6.9 ± 6.8^*∗*^	1.1 ± 1.03^*∗*^
End of observation at rest (16′ of the test)	Median (Q1, Q3)	8.4(0.04, 31.62)^*∗*^	0.5(0.15, 1.61)^*∗*^
	Average ± SD	11.1 ± 10.96^*∗*^	0.8 ± 0.6^*∗*^

LTrPs, latent trigger points; Q1 and Q3, first and third quartile; SD, standard deviation; ^*∗*^*p* < 0.05, Mann–Whitney *U* test.

## Data Availability

The data used to support the findings of this study are available from the corresponding author upon request.

## Abbreviations

TrPs: Trigger points
CS: Central sensitization
LTrPs: Latent trigger points
AURP: Autonomic referred pain
AURPT0: Autonomic referred pain with the temperature not registered for the patient before the stimulation
ROI: Region of interest
Δ$\bar{T}$°: Change in the mean temperature of the area
SD: Standard deviation
ANS: Autonomic nervous system
IRT: Infrared thermal camera/imaging.
